# A Study on the Temperature-Dependent Behavior of Small Heat Shock Proteins from Methanogens

**DOI:** 10.3390/ijms26125748

**Published:** 2025-06-16

**Authors:** Nina Kurokawa, Mima Ogawa, Rio Midorikawa, Arisa Kanno, Wakaba Naka, Keiichi Noguchi, Ken Morishima, Rintaro Inoue, Masaaki Sugiyama, Masafumi Yohda

**Affiliations:** 1 Department of Biotechnology and Life Science, Tokyo University of Agriculture and Technology, Tokyo 184-8588, Japan; nina.kurokawa@yohda.net (N.K.); ron1823212@outlook.jp (M.O.); rio.midorikawa@yohda.net (R.M.); arisa.kanno@yohda.net (A.K.); wakaba.naka@yohda.net (W.N.); 2 Instrumentation Analysis Center, Tokyo University of Agriculture and Technology, Tokyo 184-8588, Japan; knoguchi@cc.tuat.ac.jp; 3 Institute for Integrated Radiation and Nuclear Science, Kyoto University, Osaka 590-0494, Japan; morishima.ken.8e@kyoto-u.ac.jp (K.M.); inoue.rintaro.5w@kyoto-u.ac.jp (R.I.); sugiyama.masaaki.5n@kyoto-u.ac.jp (M.S.)

**Keywords:** chaperone, small heat shock protein, methanogen, analytical ultracentrifuge, stress

## Abstract

Small heat shock proteins (sHsps) are ubiquitous low-molecular-weight chaperones that prevent protein aggregation under cellular stress conditions. In the absence of stress, they assemble into large oligomers. In response to stress, such as elevated temperatures, they undergo conformational changes that expose hydrophobic surfaces, allowing them to interact with denatured proteins. At heat shock temperatures in bacteria, large sHsp oligomers disassemble into smaller oligomeric forms. Methanogens are a diverse group of microorganisms, ranging from thermophilic to psychrophilic and halophilic species. Accordingly, their sHsps exhibit markedly different temperature dependencies based on their optimal growth temperatures. In this study, we characterized sHsps from both hyperthermophilic and mesophilic methanogens to investigate the mechanisms underlying their temperature-dependent behavior. Using analytical ultracentrifugation, we observed the dissociation of sHsps from a mesophilic methanogen into dimers. The dissociation equilibrium of these oligomers was found to be dependent not only on temperature but also on protein concentration. Furthermore, by generating various mutants, we identified the specific amino acid residues responsible for the temperature dependency observed. The C-terminal region containing the IXI/V motif and the α-crystallin domain were found to be the primary determinants of oligomer stability and its temperature dependence.

## 1. Introduction

Small heat shock proteins (sHsps) are a family of molecular chaperones with relatively low molecular weights, ranging from 12 to 43 kDa [1,2], and are ubiquitously present across the biological world. Compared to other molecular chaperones, sHsps have long been underappreciated; however, recent studies have revealed their important role in maintaining intracellular proteostasis [3]. Mutations in human sHsps have been linked to various diseases, including myopathies, neuropathies, and cataracts [4]. sHsps are characterized by the presence of an α-crystallin domain, consisting of approximately 80 amino acid residues [5]. This domain, originally identified in lens crystallin proteins, adopts a β-sandwich structure in which β-strands from adjacent subunits interact to form a stable dimer. The IXI/V motif located at the C-terminus of sHsps interacts with the α-crystallin domain of neighboring dimers, thereby promoting the formation of oligomeric structures [6].

Most sHsps form large oligomeric structures composed of 12 to 36 subunits [7]. However, detailed atomic-level structural information on sHsp oligomers remains limited. Archaeal sHsps typically form spherical 24-mer oligomers with a diameter of approximately 12 nm [8,9]. In contrast, the sHsp from wheat (wHsp16.9) forms a double-ring-shaped oligomer consisting of 12 subunits [10], whereas the sHsp from the fission yeast *Schizosaccharomyces pombe*, SpHsp16.0, assembles into a hexadecameric oligomer composed of eight dimers, forming an elongated sphere with 4-2-2 symmetry [11]. Sip1, one of the 16 sHsps identified in *Caenorhabditis elegans*, exhibits chaperone activity that is regulated by pH [12]. Under acidic conditions, Sip1 inhibits protein aggregation in a concentration-dependent manner. Its oligomeric structure changes in response to pH, with 24-mer, 28-mer, and 32-mer assemblies observed by cryo-electron microscopy (cryo-EM). Among these, the crystal structure of the 32-mer has been resolved. At a low pH of 5.8, Sip1 shifts to a relatively small oligomeric form. In humans, ten sHsp genes have been identified in the genome [13]. Recently, the crystal structure of human Hsp27 (also known as HspB1) was reported [14]. This protein forms a spherical oligomeric complex composed of 24 monomers, and each monomer features a structurally conserved α-crystallin domain consisting of a six-stranded β-sandwich flanked by flexible N- and C-terminal regions. In Chinese hamsters, HspB1 exists as an 18-mer or 24-mer complex and is in dynamic equilibrium with the dissociated oligomers in the hexameric unit. The hexamer further dissociates to dimers [15].

In their large oligomeric state, sHsps typically do not exhibit chaperone activity. However, under stress conditions such as elevated temperatures, they dissociate, exposing hydrophobic regions that help prevent the irreversible aggregation of denatured proteins [16]. Nevertheless, some studies suggest that complete dissociation of oligomers is not required for the suppression of protein aggregation [17,18], and a unified mechanism for this suppression has yet to be established. In mammals, phosphorylation of serine residues in the N-terminal region allows sHsps to exhibit chaperone activity [19,20]. sHsps are found in organisms across a wide range of temperature environments, from psychrophiles to hyperthermophiles. These proteins undergo structural transitions and demonstrate functional activity at temperatures exceeding the organism’s optimal growth temperature. However, the precise mechanism by which sHsps sense and respond to temperature changes remains unclear. In the acidothermophilic archaeon *Sulfolobus tokodaii*, it has been reported that mutations in the IXI/V motif of its sHsp alter temperature-dependent behavior [21]. This study indicated that activation is associated with oligomer dissociation. However, since the IXI/V motif is conserved across all sHsps, this mechanism alone cannot fully account for the observed temperature dependence.

Methanogens are a group of microorganisms that produce methane under anaerobic conditions and are classified within the domain Archaea [22]. In anaerobic environments, organic materials are decomposed through the cooperative actions of various microorganisms. Methanogenic archaea are responsible for the final step in the degradation of organic matter, generating methane from limited substrates such as hydrogen, formate, acetate, and methylamines, which are produced by fermentative bacteria and other organisms. Methanogens are ubiquitously distributed in anaerobic habitats, including sediments of swamps and rivers, the digestive tracts of animals and insects, paddy fields, marine sediments, and anaerobic wastewater treatment reactors. They exhibit remarkable diversity in physiological characteristics, ranging from hyperthermophilic to psychrophilic and halophilic types, making them one of the most physiologically varied groups of microorganisms.

The hyperthermophilic methanogen *Methanocaldococcus jannaschii* was discovered in 1983 by the Woods Hole Oceanographic Institution at a hydrothermal vent located 2600 m deep in the East Pacific Ocean [23]. It is a slightly irregularly shaped, flagellated coccus that grows in seawater supplemented with sulfur at a slightly acidic pH, utilizing formate or hydrogen and carbon dioxide as energy sources. Notably, it has an exceptionally fast growth rate, with a doubling time of only 25 min—the fastest among known methanogens. *M. jannaschii* grows within a temperature range of 48–86 °C, with an optimal temperature of 85 °C. Its complete genome sequence was determined in 1996 [24] and contains a single gene encoding a small heat shock protein (sHsp). The 2.9 Å resolution crystal structure of the 24-subunit homo-oligomer MjHSP16.5 [8], the first structure determined for an sHsp, reveals a tripartite domain architecture that represents the prevailing features seen in the sHsp family. The N-terminal domain (NTD) of MjsHSP16.5 (residues 1–43) is buried within the cavity of the protein oligomer and is notably characterized by its richness in phenylalanine residues. With the exception of residues 36–40, which form the β1 strand, the majority of residues in the NTD are disordered but predicted to adopt a helical conformation [25]. Interestingly, MjHSP16.5 retains the ability to form native-like oligomeric assemblies, even when truncated at the NTD. A high-resolution structure of the MjHSP16.5 24-mer was also obtained using single-particle cryo-EM, allowing for comparison with the crystallographic structure. While both models depict a hollow spherical oligomer, the cryo-EM structure is believed to more closely represent the protein’s native state. Notably, the cryo-EM model revealed density corresponding to residues Thr24–Thr33 of the NTD, a region that remains unresolved in most crystal structures [26]. At temperatures above 60 °C, the subunits of MjHSP16.5 undergo rapid and reversible exchange, with the exchange reactions showing strong temperature dependence. At 37 °C, MjHSP16.5 displays significantly lower chaperone activity compared to other sHsps. Given the high sequence similarity among methanogen-derived sHsps and their markedly different temperature-dependent behaviors, this study was conducted to elucidate the molecular mechanisms underlying the temperature responsiveness of methanogenic sHsps.

## 2. Results

**Figure 1:**
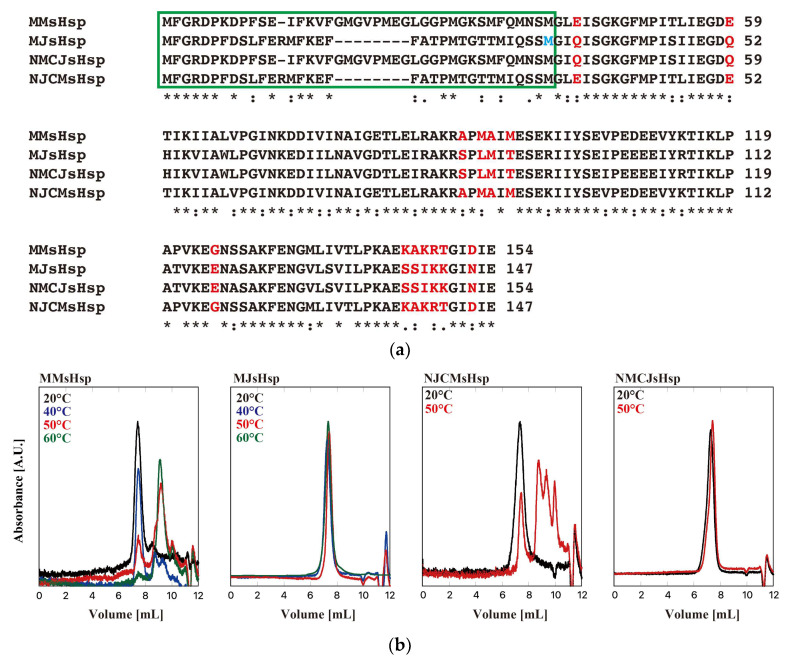
Sequence alignment and HPLC-SEC of MMsHsp, MJsHsp, NMCJsHsp, and NJCMsHsp. (**a**) Sequence alignment of MMsHsp, MJsHsp, NMCJsHsp, and NJCMsHsp. The T33M mutation of MJsHsp is demonstrated in blue. The N-terminal region is enclosed in a green box. The amino acids that were mutated in this study are marked in red. “*” indicates strictly conserved residues, “:” indicates strongly similar residues, and “.” indicates weakly similar residues. (**b**) Oligomeric structures of MMsHsp, MJsHsp, NMCJsHsp, and NJCMsHsp were analyzed by HPLC-SEC at various temperatures. A total of 50 µL of 40 µM sHsp was applied to SB-804HQ, and absorbance was monitored at 280 nm at 20 °C and at elevated temperatures (40 °C, 50 °C, and 60 °C). A.U.: arbitrary unit.

**Figure 2:**
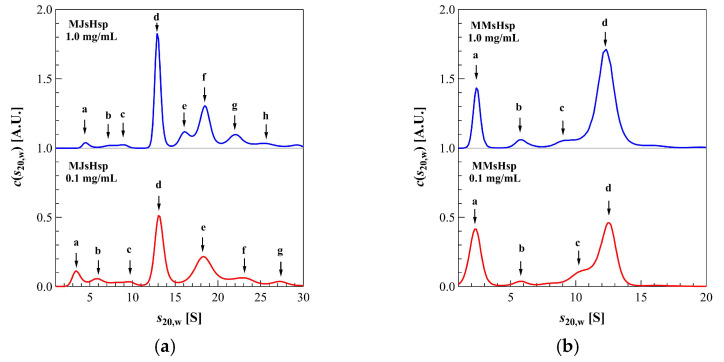
AUC results of MJsHsp (**a**) and MMsHsp (**b**) at 40 °C. Blue and red lines represent *c*(*s*_20,w_), indicating the results at 1.0 mg/mL and 0.1 mg/mL, respectively. Peaks a–h correspond to those listed in Table 1.

Figure 1 shows the sequence alignment of sHsps from *M. jannaschii* (designated as MJsHsp in this manuscript) and *Methanococcus maripaludis*, a mesophilic methanogen (designated as MMsHsp) [27]. *M. maripaludis* optimally grows at 35–40 °C, and its heat shock response is likely triggered at temperatures above 40 °C, which is much lower than the ~90 °C threshold in *M. jannaschii*. The genes encoding MJsHsp and MMsHsp were synthesized, and a point mutation was introduced into MJsHsp to substitute threonine at position 33 with methionine, enabling N-terminal sequence exchange via restriction digestion using NcoI. The genes were cloned into a pET23 expression vector, and, using the constructed plasmids, both MJsHsp and MMsHsp were expressed in *E. coli* BL21(DE3) cells. The proteins were purified via ion exchange chromatography, followed by size-exclusion chromatography. Their oligomeric states were then analyzed using high-performance liquid chromatography–size-exclusion chromatography (HPLC-SEC) at room temperature and at elevated temperatures of 50 °C and 60 °C (Figure 1). At room temperature, both MJsHsp and MMsHsp existed as large oligomeric complexes. At 50 °C, the MJsHsp oligomer remained stable, whereas MMsHsp dissociated into smaller oligomeric forms. Since the MJsHsp oligomer was stable even up to 60 °C, it was possible to conclude that the T33M mutation had a minimal impact on temperature-dependent oligomer stability. We then examined the oligomeric structures of the proteins using analytical ultracentrifugation (AUC) at 40 °C, the highest temperature suitable for AUC (Figure 2 and Table 1). MMsHsp existed primarily as a 24-mer, with minor proportions of 8-mer and 16-mer species. Upon dilution to 0.1 mg/mL, the proportion of the 24-mer decreased, while the amount of dimer increased. This result clearly demonstrates that, in addition to temperature, concentration also influences the dissociation equilibrium of sHsp oligomers. By contrast, MJsHsp predominantly existed as a 24-mer, along with aggregated large oligomers, and there was minimal dissociation into smaller oligomers. When the concentration was reduced, the formation and dissociation of larger oligomers slightly increased.

The most significant difference between MJsHsp and MMsHsp lies in the NTD. Therefore, we created mutants with swapped NTDs (Figure 1). These mutants were designated as NMCJsHsp, which is MJsHsp with the NTD of MMsHsp, and NJCMsHsp, which is MMsHsp with the NTD of MJsHsp. Contrary to our expectations, we found that the NTD was not related to the temperature dependence of oligomer stability (Figure 1). Almost identical results were obtained in the duplicate experiments.

**Figure 3:**
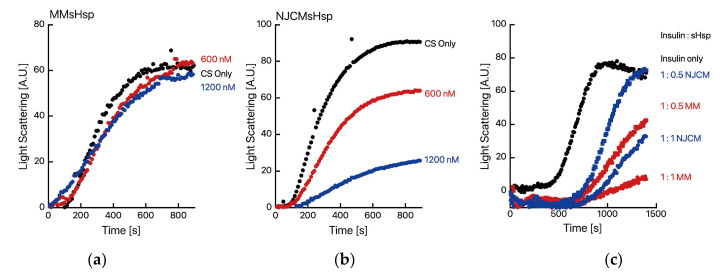
Chaperone function of MMsHsp and NJCMsHsp. The thermal aggregation of CS from a porcine heart was monitored by measuring light scattering at 500 nm with a spectrofluorometer at 50 °C. CS (50 nM, monomer) was incubated in an assay buffer with or without MMsHsp (**a**) or NJCMsHsp (**b**) at the specified concentration (600 nM and 1200 nM). (**c**) A total of 50 µM of insulin was treated with DDT, with or without MMsHsp or NJCMsHsp at the specified molar ratio. Aggregation of insulin was monitored by measuring light scattering at 360 nm with a spectrofluorometer at 30 °C.

We then investigated the citrate synthase from porcine heart (CS) aggregation suppression activity of MMsHsp and NJCMsHsp. Curiously, MMsHsp exhibited almost no CS aggregation suppression activity (Figure 3), whereas NJCMsHsp demonstrated significant aggregation suppression activity. Almost identical results were obtained in the duplicate experiments.

However, MMsHsp did demonstrate insulin aggregation suppression activity. This result suggests that the NTD plays a role in interactions with denatured proteins. As expected, MJsHsp and NMCJ could not protect CS from thermal aggregation as they did not dissociate at 50 °C (Supplementary Figure S1).

**Figure 4:**
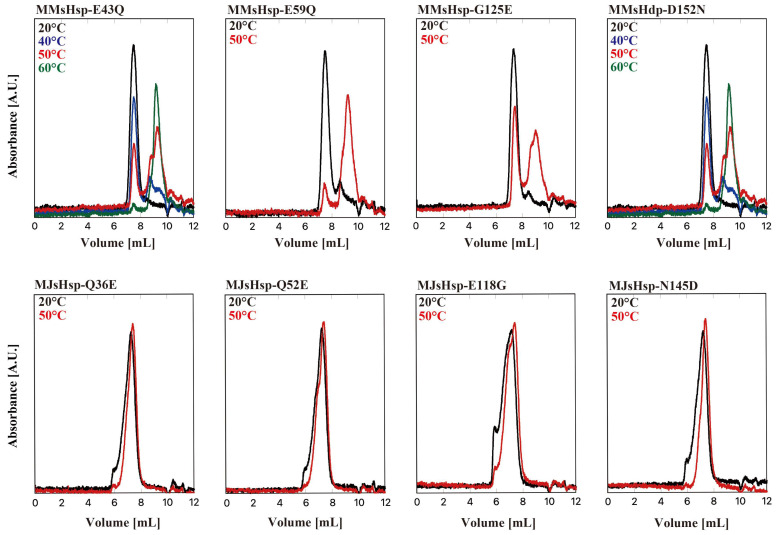
HPLC-SEC of single mutants of MMsHsp and MIsHsp. Oligomeric structures of single mutants of MMsHsp and MJsHsp were analyzed via HPLC-SEC at various temperatures. A total of 50 µL of the 40 µM sHsp variant was applied to SB-804HQ, and absorbance was monitored at 280 nm at 20 °C and at elevated temperatures (40, 50, and 60 °C).

By comparing the sequences of MJsHsp with three sHsps from mesophilic methanogens, including MMsHsp, we identified four amino acid residues that may be responsible for the temperature dependency in MJsHsp, Q36, Q52, E118, and N145, which correspond to E43, E59, G118, and D152 in MMsHsp, respectively (Figure 1). We then created mutants of MJsHsp and MMsHsp with the following amino acid substitutions: MJsHsp-Q36E, MJsHsp-Q52E, MJsHsp-E118G, MJsHsp-N145D, MMsHsp-E43Q, MMsHsp-E59Q, MMsHsp-G125E, and MMsHsp-D152N. Their temperature dependencies are shown in Figure 4. Among the four MMsHsp mutants, MMsHsp-E43Q, MMsHsp-G125E, and MMsHsp-D152N showed a slight increase in oligomer stability compared to the wild type at 50 °C, whereas MMsHsp-E59Q had almost no effect. By contrast, we did not observe oligomer dissociation in the MJsHsp mutants at 50 °C. Interestingly, the oligomer peaks were broadened, indicating partial aggregation, which may correspond to the aggregated complexes observed in the AUC.

**Figure 5:**
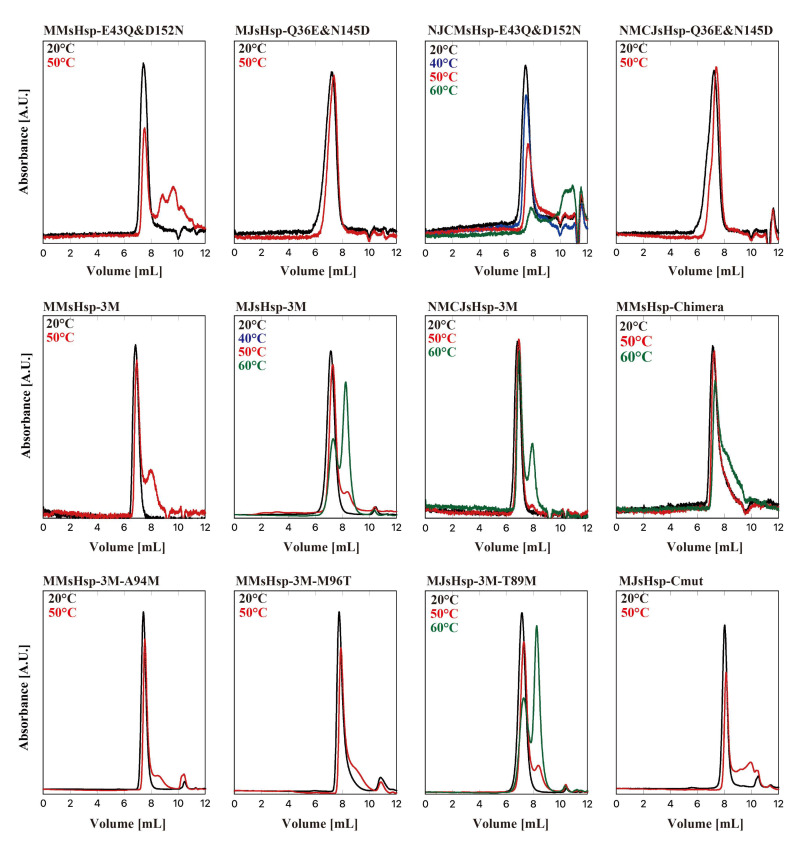
HPLC-SEC of the double, triple, and quadruple mutants, MMsHsp-Chimera and MJsHsp-Cmut. Oligomeric structures of single mutants of MMsHsp and MJsHsp were analyzed via HPLC-SEC at various temperatures. A total of 50 µL of the 40 µM sHsp variant was applied to SB-804HQ, and absorbance was monitored at 280 nm at 20 °C and at elevated temperatures (40, 50, and 60 °C).

We next examined the effects of double and triple mutations (Figure 5). The double mutation E43Q and D152N significantly improved the stability of MMsHsp. By contrast, the oligomer stability of MJsHsp at 50 °C was not affected by the corresponding double mutation (Q36E and N145D). We also applied double mutations to the N-terminal swap mutants, which revealed that the NMCJsHsp-Q36E&N145D oligomers remained stable at 50 °C. Interestingly, the peak area of NJCMsHsp-E43Q&D152N decreased at both 50 °C and 60 °C. This was likely due to nonspecific interactions with the HPLC column. Significant oligomer stabilization was observed in MMsHsp_E43Q&G125E&D152N (MMsHsp-3M). Partial dissociation of the MJsHsp-Q36E&E118GN145D (MJsHsp-3M) oligomers was observed at 50 °C, with dissociation becoming more pronounced at 60 °C. Because we observed aggregation in the MJ mutants, likely due to the exposure of hydrophobic surfaces, we used a buffer containing 20% ethylene glycol for the HPLC-SEC of MJsHsp-3M. The NMCJ-Q36E&E118G&N145D (MMCJ-3M) oligomers were stable at 50 °C but partially dissociated at 60 °C.

Although the temperature dependency changed significantly, the oligomers of MJsHsp-3M and NMCJ-3M remained stable compared to MMsHsp at 50 °C. Therefore, we attempted to identify the region responsible for the temperature dependency by constructing chimera mutants of NMCJsHsp-3M and MMsHsp-3M. The chimera mutants were constructed using the In-fusion method, incorporating the five amino acids conserved in the α-crystallin domain [24]. As shown in Supplementary Figure S2, the chimera mutant (MMsHsp-Chimera) is identical to MMsHsp-3M except for the amino acid residues between Ile-42 and Arg100, which correspond to Ile-35 and Arg-93 in MJsHsp. The oligomer of MMsHsp-Chimera exhibited higher stability at 50 °C compared to that of MMsHsp-3M, suggesting that the amino acids between Ile-35 and Arg-93 in MJsHsp also contributed to oligomer stability (Figure 5). We then compared the amino acid sequences in this region between MJsHsp and the three sHsps from mesophilic methanogens. Among the several differing residues, we focused on Met-87 and Thr-89 in MJsHsp, as these positions were substituted with amino acids containing different physicochemical properties—Ala and Met, respectively. Then, the corresponding residues in MMsHsp-3M were mutated to those of MJsHsp. The mutants, MMsHsp-3M&A94M and MMsHsp-3M&M96T, exhibited a slight increase in oligomer stability at 50 °C compared with MMsHsp-3M (Figure 5). Contrary to the expectations, the MJsHsp-3M&T89M mutant showed almost the same stability as MJsHsp-3M (Figure 5).

To investigate the effect of other amino acid residues, we compared the sequences of sHsps from various methanogens (Supplementary Figure S3). We found that the amino acid residues adjacent to the IXI/V motif were associated with temperature dependency. The C-terminal sequence of sHsps from hyperthermophilic methanogens, such as MJsHsp, is KKGINIE (residues 141–147). By contrast, sHsps from thermophilic and mesophilic methanogens, including MMsHsp, typically possess the sequence RRGINIE. Thus, to investigate the contribution of these sequences to binding affinity, we performed computational docking simulations between an MJsHsp monomer and the respective C-terminal peptides and calculated the binding free energy (ΔG) (Table 2). This analysis revealed that amino acid substitutions led to a decrease in the absolute value of ΔG. Specifically, the RRGINIE sequence from thermophilic methanogens demonstrated lower binding affinity (i.e., less negative ΔG) compared to the KKGINIE sequence from hyperthermophiles, whereas the RTGIDIE sequence from mesophilic methanogens exhibited the lowest affinity. Notably, the substitution of Lys141 and Lys142 emerged as a key factor contributing to this trend, suggesting that these residues play a critical role in regulating binding affinity. We then constructed a mutant of MJsHsp, replacing the amino acid residues adjacent to the IXI motif, designated MJsHsp-Cmut (MJsHsp-K141R&K142T&N145D), and this oligomer partially dissociated at 50 °C (Figure 5).

**Figure 6:**
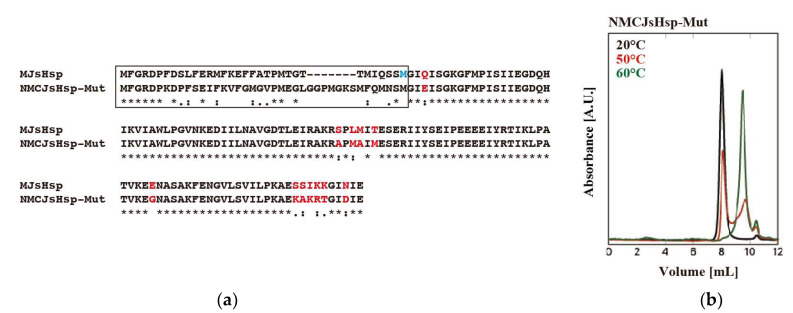
Sequence alignment and HPLC-SEC of NMCJsHsp-Mut. (**a**) Sequence alignment of NMCJsHsp-Mut and NJsHsp. T33M mutation of MJsHsp is shown in blue. The mutated amino acids of MCJsHsp-Mut are marked in red. “*” indicates strictly conserved residues, “:” indicates strongly similar residues, and “.” indicates weakly similar residues. The N-terminal region is indicated by a black box. (**b**) Oligomeric structures of NMCJsHsp-Mut were analyzed via HPLC-SEC. A total of 50 µL of 40 µM of NMCJsHsp-Mut was applied to SB-804HQ, and absorbance was monitored at 280 nm at 20 °C and at elevated temperatures (40 °C, 50 °C, and 60 °C).

Lastly, upon deciding to combine various mutations, we used NMCJsHsp due to the issues caused by the hydrophobic nature of the MJsHsp N-terminus. The constructed mutant, NMCJsHsp-Mut, contains amino acid mutations of Q36E, S84A, L86M, M87A, T89M, E118G, S138K, S139A, I140K, K141R, K142T, and N145D (the numbers correspond to those of MJsHsp), in addition to an N-terminus replacement. NMCJsHsp-Mu exhibited significant oligomer dissociation at 50 °C compared to MJsHsp-3M&T89M. At 60 °C, its oligomer completely dissociated into small oligomers (Figure 6).

## 3. Discussion

The X-ray crystal structure of MjHSP16.5 (MJsHsp) has previously been determined, excluding the NTD. The dimer serves as the structural unit, with the IXI motif in the C-terminal domain inserted into the groove between two β-strands of an adjacent dimer. Although the structure of MmHsp (MMsHsp) has yet to be experimentally resolved, AUC analysis indicates that, similar to MJsHsp, MMsHsp also forms a 24-mer oligomer. Furthermore, the monomer structure predicted by AlphaFold3 closely resembles that of MJsHsp. These findings suggest that MMsHsp likely adopts a structure nearly identical to that of MJsHsp. Treatment of the sedimentation velocity (SV)-AUC data obtained for a mixture of different oligomeric forms in a dynamic equilibrium (a self-interacting system) using the approach devised for a mixture of non-interacting molecules can lead to the inaccuracy in estimation of the values of the sedimentation coefficients and masses. However, the molecular mass calculated from the SV-AUC data of the main peak of each sHsp corresponds to the expected 24-mer derived from the structure, suggesting that the in-fluence of dynamic equilibrium is minimal.

Unexpectedly, the N-terminal region—where the sequences of MJsHsp and MMsHsp differ the most—had little to no effect on oligomer stability. Since the N-terminal region adopts a largely disordered conformation within the oligomer, it is not directly involved in oligomer formation. A previous study demonstrated that an N-terminal deletion mutant adopts a structure nearly identical to that of the wild type [28].

In the crystal structure of MjHsp16.5 (MJsHsp), Q36 is positioned in close proximity between adjacent dimers (Supplementary Figure S4). We hypothesize that substituting this residue with glutamic acid introduces electrostatic repulsion, thereby destabilizing the oligomer. N145 and E118 in MJsHsp are located at the interaction site of the C-terminal IXI motif, a region known to be critical for oligomer assembly (Supplementary Figure S5). We consider that mutations at this site may disrupt the interaction between the IXI motif and the adjacent dimer. S138, S139 I140, K141, and K142 are also located close to the C-terminus and seem to interact with the adjacent dimer (Supplementary Figure S6).

In the crystal structure of MJsHSP16.5, S84, L86, M87, and T89 are located in a loop region within the α-crystallin domain. This loop lies within the dimer interface and is believed to interact with the β-strand of the adjacent monomer (Supplementary Figure S7), making this loop region important for maintaining the stability of the oligomeric structure.

MJsHsp has been demonstrated as undergoing subunit exchange. Although the efficiency of subunit exchange at 40 °C is expected to be low, exchange is still thought to occur at a certain rate. In these cases, the N-terminal region is temporarily exposed, which could lead to the formation of aggregates. In contrast, MMsHsp does not form such aggregates due to its lower hydrophobicity at the N-terminal. It is likely that MJsHsp adapts to high temperatures, and, in a high-temperature environment with intense molecular motion, hydrophobic interactions with denatured proteins are strengthened. Additionally, the formation of large oligomers by MJsHsp is thought to result from the exposure of hydrophobic sites when the molecule is partially dissociated.

In this study, we found that the amino acids involved in temperature dependence were primarily located in regions associated with the interaction between the C-terminal region containing the IXI/V motif and the α-crystallin domain. The only exception was Q36 in MJsHsp. In contrast, the N-terminal region, which exhibited the greatest variation in amino acid sequence, was not associated with structural stability. These findings suggest that, in other microbial sHsps, temperature dependence is likely determined by the interaction between the C-terminal region containing the IXI/V motif and the α-crystallin domain.

## 4. Materials and Methods

### 4.1. Creation of Genes for Wild-Type and Mutant sHsps

The genes for MJsHsp, MMsHsp, MJsHsp-Cmut, and NMCJsHsp-mut were synthesized and codon-optimized for expression in *E. coli*. The nucleotide and amino acid sequences of MJsHsp and MMsHsp are shown in Supplementary Figure S8. To facilitate the construction of the NTD exchange variant, an NcoI site was introduced, resulting in the replacement of Thr-33 with Met in MJsHsp. Additionally, point mutations were introduced via DpnI-mediated, site-directed mutagenesis [29]. In-fusion method was used to generate the chimeras [30], with the primers used shown in Supplementary Table S1. The genes of wild-type and mutant sHsp were cloned into a pET23 vector and expressed in *E. coil* BL21(DE3). The expressed sHsps were purified via anion exchange chromatography, using DEAE-650 and TOYOPEARL SuperQ-650 (Tosoh Corporation, Tokyo, Japan), and gel filtration chromatography, using Superdex 200 (Cytiva, Marlborough, MA, USA). Glycerol was added to the samples to a final concentration of 20%; the samples were frozen in liquid nitrogen and stored at −30 or −80 °C.

### 4.2. HPLC-SEC

HPLC-SEC was performed using a gel filtration column (SB-804HQ, Showa Denko, Tokyo, Japan) via an HPLC system, PU-1580i (JASCO Corporation, Tokyo, Japan), connected to an MD1515 multiwavelength detector or UV/Vis detector UV4075, as described previously [21]. The sHsp variants were heated at a specified temperature for 30 min. A total of 50 µL of 40 µM preincubated sHsp was loaded onto a column heated at the same temperature and eluted with buffer B, with or without 20% ethylene glycol at a flow rate of 0.5 mL/min. Protein absorbance was monitored at 220 nm or 280 nm.

### 4.3. AUC Measurements

AUC measurements were carried out using ProteomeLab XL-I (Beckman Coulter, Brea, CA, USA), and samples were filled in 12 mm pathlength aluminum double-sector centerpieces. All measurements were performed in sedimentation velocity mode using Rayleigh interference optics at a 40,000 rpm rotor speed. The time evolution of the sedimentation data was analyzed via the multi-component Lamm formula. Then, the weight concentration distribution of the components, *c*(*s*_20,w_), was obtained as a function of the sedimentation coefficient. Here, the sedimentation coefficient was normalized to be the value at 20 °C in pure water, *s*_20,w_. The molecular mass, *M*, and association number, *N*, of each component were calculated using the following equation:

(1) $$M={\left[\frac{6\pi \eta N_{A}}{\left(1-\rho \bar{v}\right)}\right]}^{1.5}{\left(\frac{3\bar{v}}{4\pi N_{A}}\right)}^{0.5}{\left(\frac{f}{f_{0}}\right)}^{1.5}{s_{20,w}}^{1.5}\;,\;$$

(2) $$N=\frac{M}{M_{1}}\;,\;$$

where ρ, η, $\bar{v}$,$\;N_{A}$, $f/f_{0}$, and $M_{1}$ are the solvent density of pure water at 20 °C, solvent viscosity of pure water at 20 °C, partial specific volume, Avogadro number, friction ratio, and molecular mass of a monomer, respectively. These calculations were performed using SEDFIT software version 16.1c [31], and the density and viscosity of the solvents were measured with a DMA4500M density meter (Anton Paar, Graz, Austria) and a Lovis 2000 M/ME (Anton Paar, Graz, Austria) viscometer, respectively. Almost the same results were obtained by the experiment at a 60,000 rpm rotor speed.

### 4.4. Protein Aggregation Measurements

The thermal aggregation of CS was monitored by measuring light scattering at 500 nm with a spectrofluorometer (FP-6500, JASCO, Tokyo, Japan) at 50 °C, as described previously [15]. Native CS (50 nM, monomer) was incubated in TKM buffer (50 mM Tris-HCl, pH 7.5, 100 mM KCl, and 25 mM MgCl_2_) with or without an sHsp. The assay buffer was then preincubated at 50 °C and continuously stirred throughout the measurement.

The activity of inhibiting insulin aggregation was evaluated by measuring the time-dependent change in absorbance at 360 nm using a spectrofluorometer (FP-6500, JASCO Corporation, Tokyo, Japan) equipped with a thermostatted cell. First, insulin was dissolved in a buffer (25 mM HCl, 100 mM NaCl) to a concentration of 2 mg/mL. Then, 2× buffer (sodium phosphate (pH 6.8), 300 mM NaCl, 0.5 mM EDTA) was added to a four-sided quartz cell, and the sample was added in a mass ratio (insulin/sHsp = 1:0.5, 1:1) and pre-incubated for 10 min at 30 °C. Afterward, DTT was added to achieve a final concentration of 20 mM, and insulin was added to reach a final concentration of 50 µM. Absorbance at 360 nm was measured for 1400 s; during the measurement, the cell was stirred at 250 rpm and the reaction temperature maintained at 30 °C.

### 4.5. Monomer–C-Terminal Domain Docking Simulation

The three-dimensional structural data for MJsHsp were obtained from the RCSB Protein Data Bank (www.rcsb.org) with the PDB code entry 4I88. Monomer and C-terminal residues from positions 141 to 147 were extracted using PyMOL2 version 3.0.3. Subsequently, amino acid residues within the C-terminal peptide were substituted as needed. The three-dimensional structural data of the extracted monomer and C-terminal peptide were prepared using AutoDockTools by adding hydrogen atoms and computing Gasteiger charges. Docking of the MJsHsp monomer with the C-terminal peptide (residues 141–147) was performed using the empirical free energy function and the Iterated Local Search global optimization algorithm implemented in AutoDock vina [32]. The grid box for the docking process was established using AutoDockTools, with grid dimensions of 40 Å × 40 Å × 40 Å, centered between the beta sheets around positions 70 and 120 of the monomer. A spacing of 0.375 Å was used. The docking parameters included an exhaustiveness value of 8, with 100 binding modes (num_modes) generated and an energy range of 3 kcal/mol. The binding pose and binding free energy ΔG (kcal/mol) of the lowest energy binding mode were also calculated.

## Figures and Tables

**Table 1:** Parameters obtained by AUC measurements.

**MMsHsp**						
***c* [mg/mL]**	***f*/*f*_0_**	**Peak**	***s*_20,w_ [S]**	***M* [kDa]**	***N***	***w* [%]**
1.0	1.46	a	2.37	34	2	16.8
		b	5.81	132	8	4.6
		c	9.72	285	17	5.3
		d	12.33	408	24	73.3
0.1	1.43	a	2.25	31	2	31.3
		b	5.81	128	8	4.2
		c	10.31	303	18	10.9
		d	12.44	402	24	53.6
**M** **JsHsp**						
***c* [mg/mL]**	***f*/*f*_0_**	**Peak**	***s*_20,w_ [S]**	***M* [kDa]**	***N***	***w* [%]**
1.0	1.37	a	4.57	82	5	2.1
		b	7.62	176	11	1.7
		c	8.97	225	14	2.0
		d	12.87	386	23	38.7
		e	16.08	540	33	8.8
		f	18.45	663	40	27.6
		g	22.01	864	52	12.1
		h	25.39	1071	65	7.0
0.1	1.36	a	3.39	52	3	7.3
		b	5.76	114	7	5.7
		c	9.48	242	15	4.8
		d	13.04	391	24	35.7
		e	18.28	648	39	30.7
		f	23.02	916	55	10.6
		g	27.26	1179	71	5.2

*c*: concentration; *f*/*f*_0_: friction ratio; *s*_20;w_: reduced sedimentation coefficient in pure water at 20 °C; *M*: molecular mass; *N*: association number; *w*: weight fraction.

**Table 2:** Interaction energy between C-terminus and α-crystallin, calculated using AutoDock vina version 1.2.3.

sHsp	Mutation	Sequence	ΔG (Kcal/mol)	ΔΔG
Hyperthermophilic (MJsHsp)	WT	KKGINIE	−12.87	-
Mutant 1	N145D	KKGIDIE	−12.81	0.059
Mutant 2	K142T	KTGINIE	−12.65	0.223
Mutant 3	K141R	RKGINIE	−12.53	0.346
Thermophilic	K141R/K142R	RRGINIE	−12.68	0.189
Mesophilic (MMsHsp)	K141R/K142T/N145D	RTGIDIE	−12.41	0.458

## Data Availability

Data is contained within the article and Supplementary Materials.

## Supplementary Materials

The following supporting information can be downloaded at: https://www.mdpi.com/article/10.3390/ijms26125748/s1.

## Abbreviations

MJsHsp	sHsp of *Methanocaldococcus jannaschii* with T33M mutation
MMsHsp	sHsp of *Methanococcus maripaludis*
HPLC-SEC	High-performance liquid chromatography–size-exclusion chromatography
AUC	Analytical ultracentrifugation
CS	Citrate synthase from porcine heart
NMCJsHsp	MJsHsp with the N-terminal domain of MMsHsp
NJCMsHsp	MMsHsp with the N-terminal domain of MJsHsp
MJsHsp-Q36E	MJsHsp with Q36E mutation
MJsHsp-Q52E	MJsHsp with Q52E mutation
MJsHsp-E118G	MJsHsp with E118G mutation
MJsHsp-N145D	MJsHsp with N145D mutation
MMsHsp-E43Q	MMsHsp with E43Q mutation
MMsHsp-E59Q	MMsHsp with E59Q mutation
MMsHsp-G125E	MMsHsp with G125E mutation
MMsHsp-D152N	MMsHsp with D152N mutation
MMsHsp-E43Q&D152N	MMsHsp with E43Q and D152N mutations
MJsHsp-Q36E&N145D	MJsHsp with Q36E and N145D mutations
NJCMsHsp-E43Q&D152N	NJCMsHsp with E43Q and D152N mutations
NMCJsHsp-Q36E&N145D	NMCJsHsp with Q36E and N145D mutations
MMsHsp-3M	MMsHsp with E43Q, G125E and D152 mutations
MJsHsp-3M	MJsHsp with Q36E, E118G and N145D mutations
NMCJsHsp-3M	NMCJsHsp with Q36E, E118G and N145D mutations
MMsHsp-Chimera	A chimera sHsp of NMCJsHsp-3M and MMsHsp-3M
MMsHsp-3M-A94M	MMsHsp with E43Q, A94M, G125E and D152 mutations
MMsHsp-3M-M96T	MMsHsp with E43Q, M96T, G125E and D152 mutations
MJsHsp-3M-T89M	MJsHsp with Q36E, T89M and N145D mutations
MJsHsp-Cmut	MJsHsp with K141R, K142T and N145D mutations
NMCJsHsp-Mut	NMCJsHsp with Q36E, S84A, L86M, M87A, T89M, E118G, S138K, S139A, I140K, K141R, K142T, and N145D mutations
A.U.	Arbitrary unit
