# Supplementary material for: A Study on the Temperature-Dependent Behavior of Small Heat Shock Proteins from Methanogens
Source: Int J Mol Sci. 2025 Jun 16;26(12):5748. doi: 10.3390/ijms26125748 (PMC12193508; doi:10.3390/ijms26125748)
Supplement: Supplementary file 1 [file ijms-26-05748-s001.zip › SupplemaryFiguresRev.pdf]

Supplementary Fig. S1

CS aggregation assay

MJsHsp

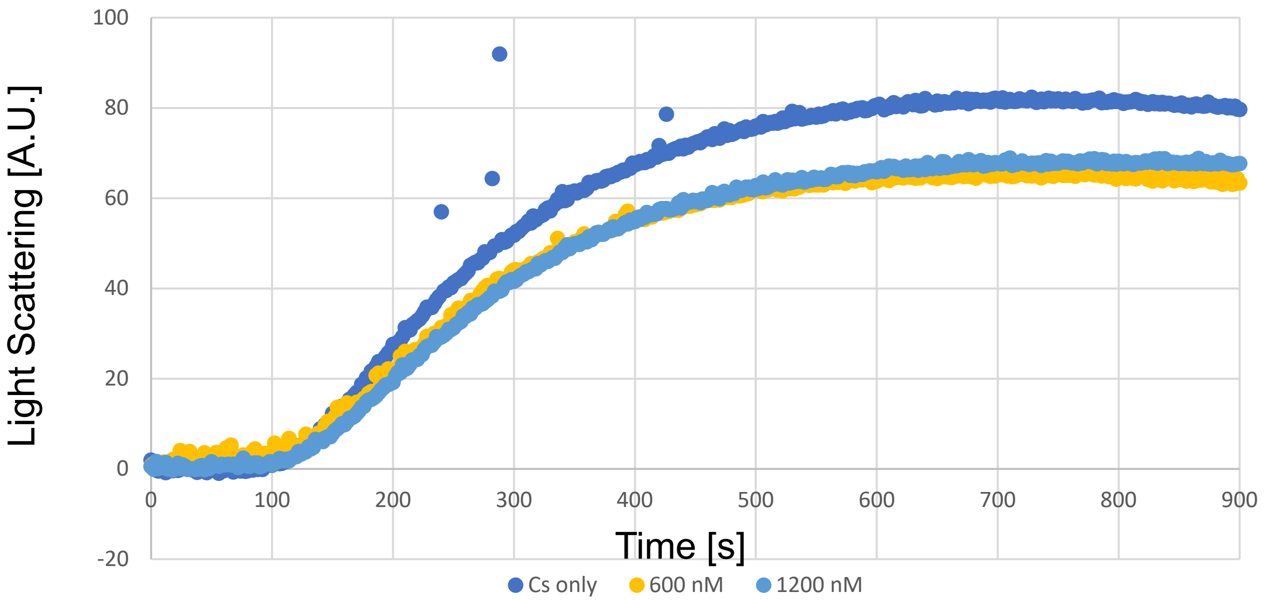

NMCJsHsp

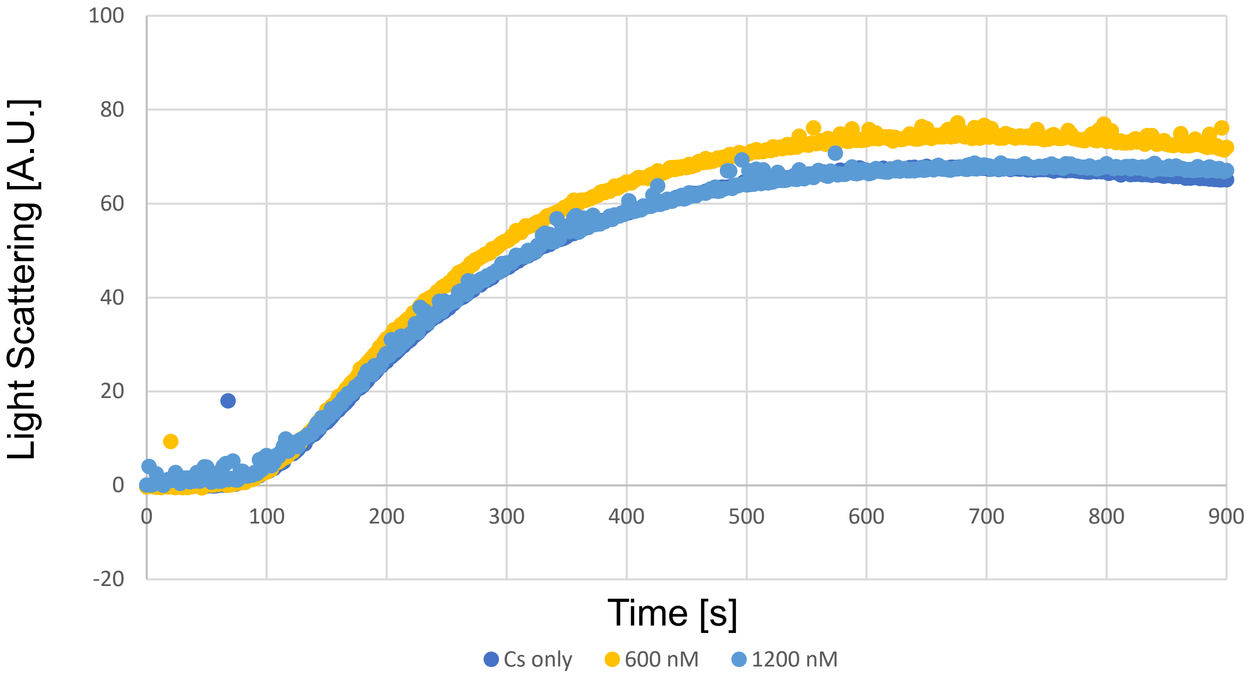

Supplementary Fig. S2

```
MMsHsp_3M          MFGRDPKDPFSEIFKVFGMGVPMEGLGGPMGKSMFQMNSMGLQISGKGFMPITLIEGDET 60
MMsHsp-Chimera     MFGRDPKDPFSEIFKVFGMGVPMEGLGGPMGKSMFQMNSMGIEISGKGFMPISIIEGDQH 60
NMCJsHsp-3M        MFGRDPKDPFSEIFKVFGMGVPMEGLGGPMGKSMFQMNSMGIEISGKGFMPISIIEGDQH 60
                   *****************************************::*********::****: 

MMsHsp_3M          IKIIALVPGINKDDIVINAIGETLELRAKRAPMAIMESEKIIYSEVPEDEEVYKTIKLPA 120
MMsHsp-Chimera     IKVIAWLPGVNKEDIILNAVGDTLEIRAKRSPLMITESERIIYSEVPEDEEVYKTIKLPA 120
NMCJsHsp-3M        IKVIAWLPGVNKEDIILNAVGDTLEIRAKRSPLMITESERIIYSEIPEEEEIYRTIKLPA 120
                   **:** :**:**:**::**:*:***:****:*: * ***:*****:**:**:*:******

MMsHsp_3M          PVKEENSSAKFENGMLIVTLPKAEKAKRTGINIE  154
MMsHsp-Chimera     PVKEENSSAKFENGMLIVTLPKAEKAKRTGINIE  154
NMCJsHsp-3M        TVKEGNASAKFENGVLSVILPKAESSIKKGIDIE  154
                    *** *:*******:* * *****.: :.**:**
```

Supplementary Fig. S3

				Growth Temperature
Methanocaldococcus jannaschii	WP_010869783.1:1-147	MFGRDPFDSLFERMFKE----FFATPMTGTTMIQSS-		85
Methanocaldococcus bathoardescens	WP_048202210.1:1-147	MFGRDPFDSLFERMLKE----FFTTPMTGTTMIQSS-		80
Methanocaldococcus vulcanius	WP_015732743.1:1-147	MIGRDPFDSLFERMFKE----LFTTPMAGTTMIQSS-		80
Methanocaldococcus fervens	WP_015791413.1:1-147	MFGRDPFDSLFERMFKE----FFTTPIAGTTMIQSS-		85
Methanocaldococcus lauensis	CAB3287543.1:1-147	MFGRDPFDSLFERMFKE----FFATPISGSTVVQSS-		80-85
Methanocaldococcus villosus	WP_004590579.1:1-148	MFGRDPFDSLFERMFRE----FFSMPMGSSTTIMQT-		80
Methanocaldococcus infernus	WP_013099762.1:1-146	MFGRDPFDSIFERMFKE----FM-MPMGGATTMISS-		85
Methanotorris formicicus		MFGRDPFSEIEKMMMEM----FMT-PFGASRM-TYK----------IGGGTSIEVSGKGF	44	75
Methanotorris igneus		MFGRDPFAEIEKMMMEM----FMT-PFTTSRM-SYR----------MGGGTSIEISGKGF	44	88
Methanothermococcus okinawensis		MFGRDPFSEIEKLMSEM----FMS-PMAGMRT-MK-----------TMSSGLEISGKGY	42	60-65
Methanofervidicoccus abyssi		MFGRDPFSEIEKLMAEM----FMT-PMMGLSS-RR-----------TITSTGLEISGKGY	43	70
Methanococcus aeolicus		MIGKDPFFE--KILSEF----LGGAPMSMTTM-TSM----------SSMGSGVEISGKGQ	43	46
Methanococcus maripaludis		MFGRDPKDPFSEIFKVF----GMGVPMEGFGGPMGK-------SMFQMSSLGLEISGKGF	49	35-39
Methanococcus vannielii		WMIGRDPKDPFSEIFKMF----GMSFPMEGFGGPMTR-------TMFQMGTAGLEISGKGY	4[illegible]	36-40

Methanocaldococcus jannaschii	MPI-SIIEGDQHIKVIAWLPGVNKEDIILNAVGDTLEIRAKR-SPLMITESERIIYSEIP	10[illegible]	85
Methanocaldococcus bathoardescens	MPI-SIIEGDKHIKVIAWLPGVNKEDIVLNAIGDTLEIRAKR-SPLMITESERIIYSEIP	10[illegible]	80
Methanocaldococcus vulcanius	MPI-SIIEGDQHIKVIAWLPGVNKEDIILNAVGDTLEIRAKR-SPLMITESERIIYSEIP	10[illegible]	80
Methanocaldococcus fervens	MPI-SIIEGDKHIKVIAWLPGVNKEDIVLNAIGDTLEIRAKR-SPLMITESERIIYSEIP	10[illegible]	85
Methanocaldococcus lauensis	MPI-SIIEGDDHIKVIAWLPGVNKEDIVLNAVGDTLEIRAKR-SPLMITESERIIYSEIP	10[illegible]	80-85
Methanocaldococcus villosus	MPI-SIIEGDDHLKVIAWLPGVNKEDIVLNAVGDSLEIRAKR-SPLMITESERIIYSEIP	10[illegible]	80
Methanocaldococcus infernus	MPI-SIIEGDEHIKVIAWLPGVAKEDIVVNAVGDTLEIRAKR-SPLMVTESERIIYSEIP	99	85
Methanotorris formicicus	MPI-TVIEGDEHVKVIAMLPGVNKEDIVLNAVGDTLEIRAKR-QPLMITESERIIYSEIP	10[illegible]	75
Methanotorris igneus	MPV-AIIEGDEHIKVVAMLPGVNKEDIVLNAVGDTLEIRAKK-APLMITESERVVYSEIP	10[illegible]	88
Methanothermococcus okinawensis	MPI-TLIEGDNNVKVIAMIPGVNKEDIVVNAVGDTLEIRAKR-APLMITESERIVYSEIP	10[illegible]	60-65
Methanofervidicoccus abyssi	MPI-TLIEGDNNIKVIAMLPGVNKEDIVLNAIGDTLEIRAKR-APLMVTESERVIYSEIP	10[illegible]	70
Methanococcus aeolicus	MPI-TLIEGDNHIKIIAMVPGIAKEDIVINAIGDTLEIRAKR-TPLMITESERIIYSEIP	10[illegible]	46
Methanococcus maripaludis	MPI-TLIEGDETIKIIAMAPGVNKNDIVINAIGETLELRAKR-APMAIMESEKVIYSEVP	10[illegible]	35-39
Methanococcus vannielii	MPL-TIIEGDESIKIIALIPGVNKSDIVINAVGDTLELRAKK-APLAIMESEKIIYSEVA	10[illegible]	36-40
	. : : . . * **. *..: : .. :.: *: ..: : :		

Methanocaldococcus jannaschii	EEE-EIYRTIKLPATVKEENASAKFENGVLSVILPKAESSIKKGINIE	147	85
Methanocaldococcus bathoardescens	EEE-EIYRTIKLPANVKEENASAKFENGVLSVTLPKAESSIKKGINIE	147	80
Methanocaldococcus vulcanius	EDE-EIYRNIKLPATVKEENASAKFENGVLSVILPKAESSIKKGINIE	147	80
Methanocaldococcus fervens	EEE-EVYRTIKLPAVVKEEDASAKFENGVLSVILPKAETSIKKGINIE	147	85
Methanocaldococcus lauensis	EEE-EVYRTIKLPAHVKEENASAKFENGVLVVTLPKAETSIKKGINIE	147	80-85
Methanocaldococcus villosus	EEE-EIYRNIKLPAVVKEDKASAKFENGVLTVILPKAESSRRKGIRID	148	80
Methanocaldococcus infernus	EEE-EVYRQIKLPSAVKEDKASAKFENGVLYITLPKVEGSRKRGIHIE	146	85
Methanotorris formicicus	EDE-EIYRTIKLPATVKEENASAKFENGMLIATLPKAEKSIKKGINIE	149	75
Methanotorris igneus	EDE-ELYRTIKLPAAVKEENASAKFENGLLIVTLPKAEKSVKKGINIE	149	88
Methanothermococcus okinawensis	EDE-EIYRTIKLPATVKEDNASAKFENGMLIVELPKSEVSIKKGINIE	147	60-65
Methanofervidicoccus abyssi	EDE-EVYRIIKLPATVKEDAASAKYENGLLIVNLPKTEASIKRGINIE	148	70
Methanococcus aeolicus	EDE-EIYRTIKLPATVKEDSAKAKYENGILIVELPKSEISIKKGIDIE	148	46
Methanococcus maripaludis	EDE-EIYKTIKLPAPVKEGNSSAKFENGMLIVTLPKAEKAKRTGIDIE	154	35-39
Methanococcus vannielii	EDE-EIYKTIKLPAHVKEGKSSAKFENGILTIALPKTEKSLRTGIDIE	154	36-40
	: . : : *. .*:* *		

RED: Hyperthermophilic

Black: Thermophilic

Blue: Mesophilic

Supplementary Fig. S4

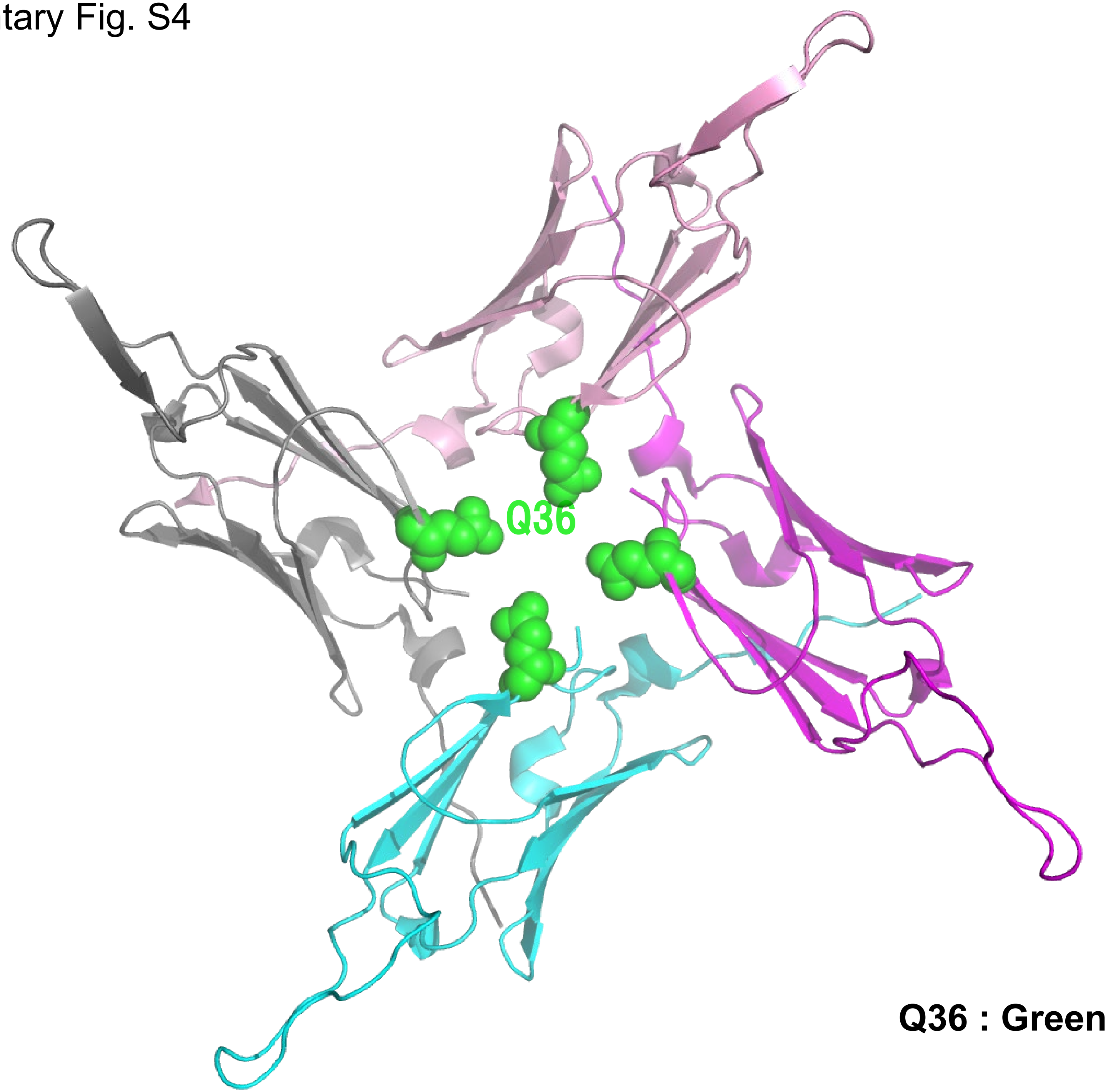

Supplementary Fig. S5

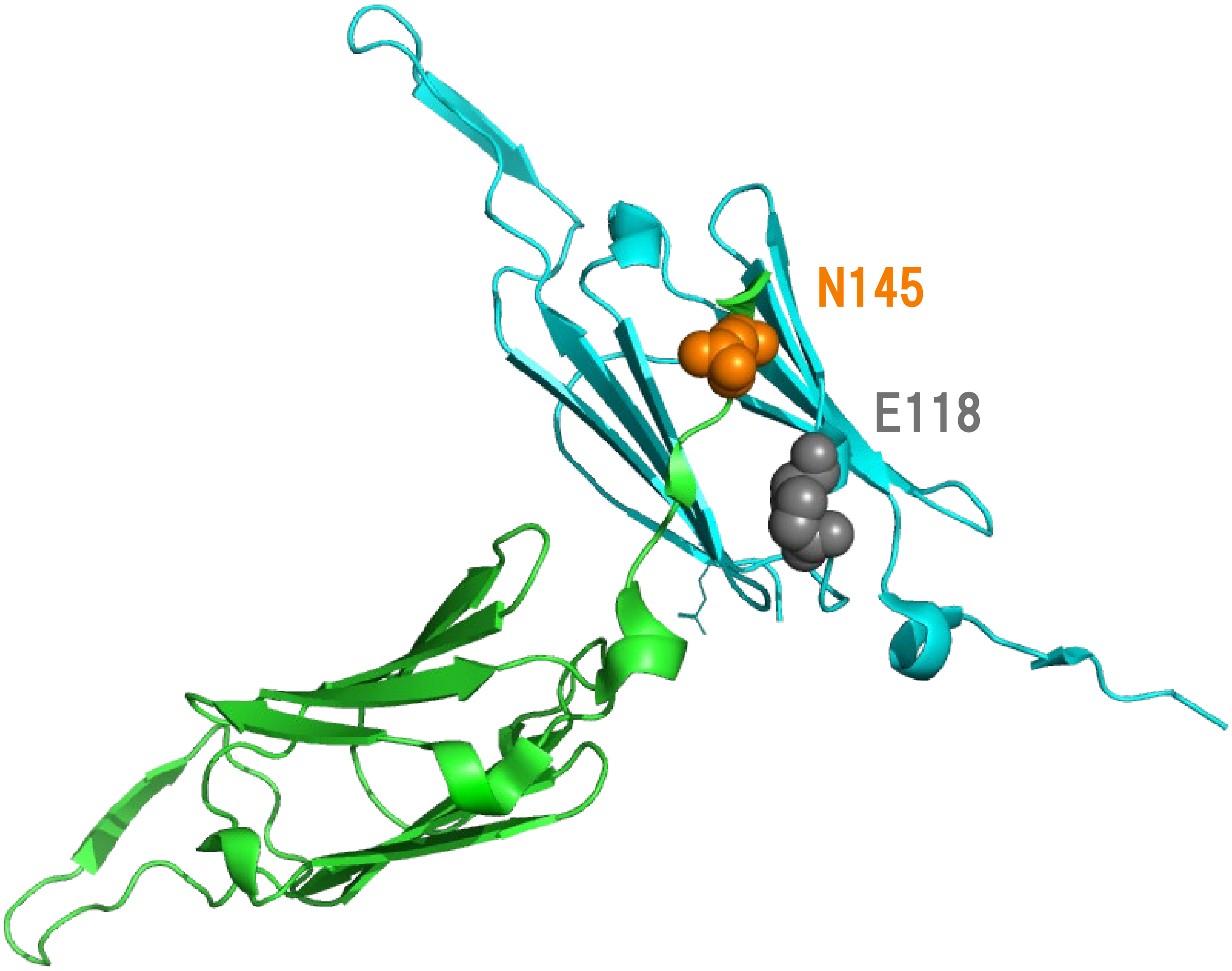

E118 : Gray, N145 : Orange

Supplementary Fig. S6

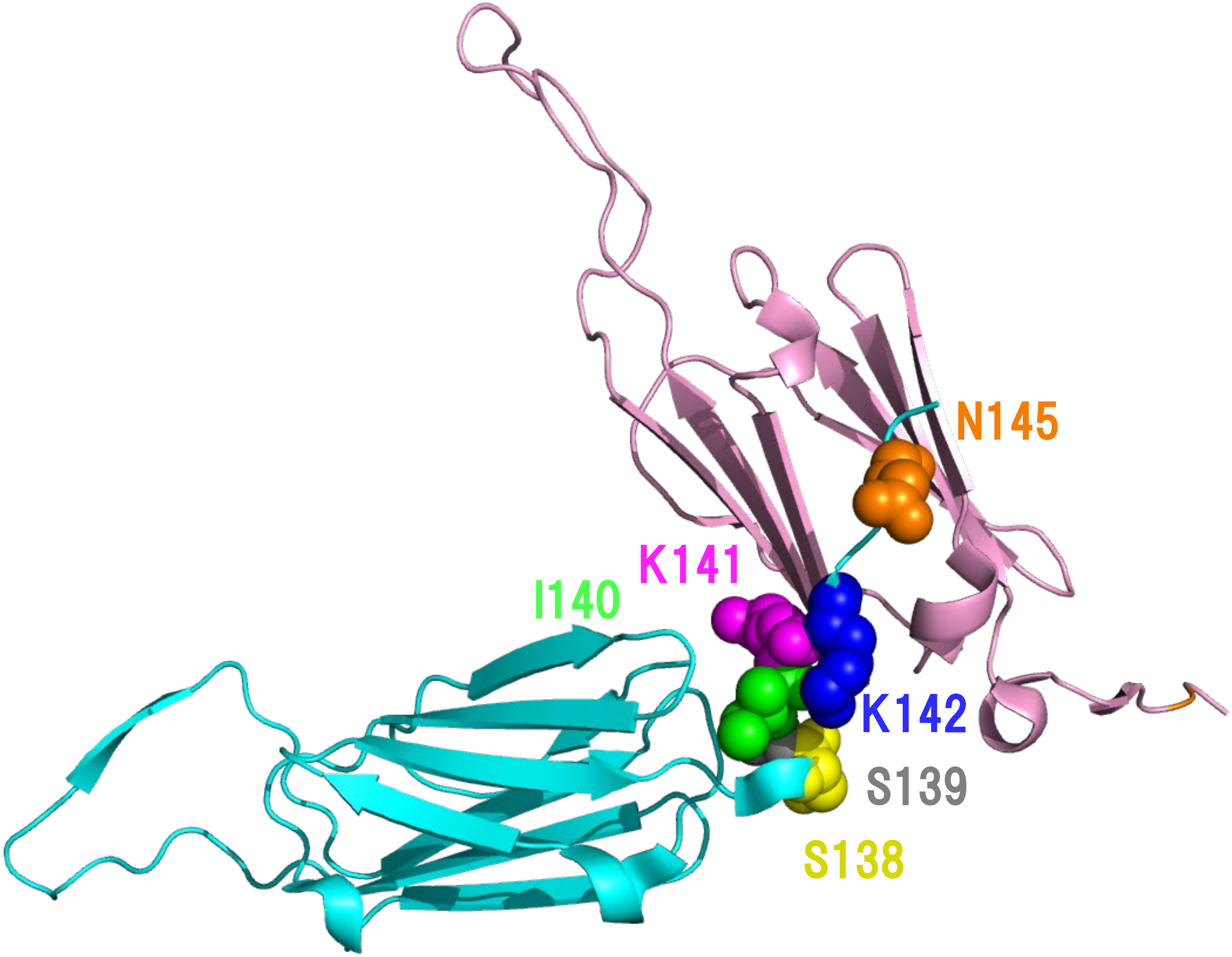

S138 : Yellow, S139 : Gray, I140 : Green, K141 : Magenta, K142 : Blue, N145 :Orange

Supplementary Fig. S7

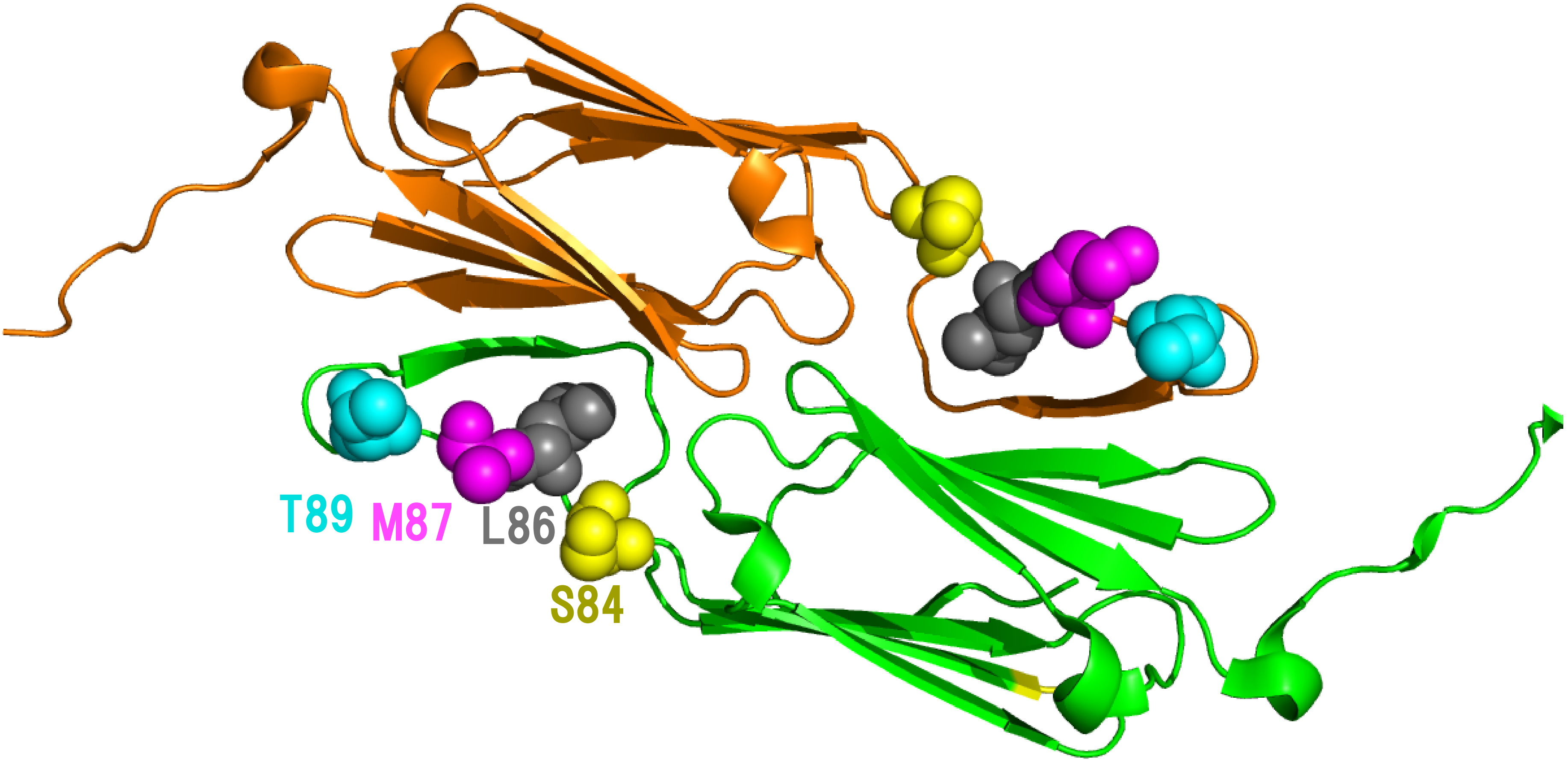

S84 : Yellow, L86 : Gray, M87 : Magenta, T89 : Cyan
