# Supplementary material for: A Study on the Temperature-Dependent Behavior of Small Heat Shock Proteins from Methanogens
Source: Int J Mol Sci. 2025 Jun 16;26(12):5748. doi: 10.3390/ijms26125748 (PMC12193508; doi:10.3390/ijms26125748)
Supplement: Supplementary file 1 [file ijms-26-05748-s001.zip › SupplementaryFigS8.pdf]

Supplementary Fig. S8

DNA and amino acid sequences of MMsHsp and MJsHsp

MMsHp

ATGTTTGGACGTGATCCGAAAGACCCGTTTTCGGAAATCTTCAAAGTCTTTGGCATGGGTGTACCAATGGAGGGTTTAGGTGGTCCGATG
M F G R D P K D P F S E I F K V F G M G V P M E G L G G P M

GGCAAAAGCATGTTCCAGATGAACTCCATGGGGCTGGAGATTAGTGGCAAAGGCTTTATGCCGATTACCCTGATTGAAGGCGATGAAACC
G K S M F Q M N S M G L E I S G K G F M P I T L I E G D E T

ATTAAGATCATTGCACTGGTTCCGGGTATCAACAAGGATGACATCGTGATTAACGCGATTGGCGAAACGCTCGAATTGCGTGCTAAACGC
I K I I A L V P G I N K D D I V I N A I G E T L E L R A K R

GCACCTATGGCGATCATGGAATCGGAGAAAATCATCTATAGCGAAGTTCCGGAAGATGAAGAAGTCTACAAGACCATCAAGCTGCCTGCT
A P M A I M E S E K I I Y S E V P E D E E V Y K T I K L P A

CCAGTGAAAGAAGGGAATTCATCCGCGAAATTCGAGAATGGAATGCTGATTGTGACTCTTCCCAAAGCCGAGAAAGCCAAACGCACAGGC
P V K E G N S S A K F E N G M L I V T L P K A E K A K R T G

ATTGACATTGAATAA
I D I E *

MJsHsp

ATGTTTGGTCGCGATCCGTTTGACAGCCTGTTTGAACGGATGTTCAAGGAATTCTTTGCAACTCCGATGACTGGAACCACGATGATCCAA
M F G R D P F D S L F E R M F K E F F A T P M T G T T M I Q

TCCTCcAtgGGTATTCAGATTTCGGGTAAAGGCTTTATGCCGATTAGCATCATTGAAGGCGATCAGCATATCAAAGTGATTGCGTGGTTA
S S M G I Q I S G K G F M P I S I I E G D Q H I K V I A W L

CCAGGGGTTAACAAAGAGGACATCATTCTGAACGCTGTTGGGGATACCTTGGAGATTCGTGCCAAACGTTCTCCGCTCATGATTACGGAA
P G V N K E D I I L N A V G D T L E I R A K R S P L M I T E

AGTGAACGCATCATTTACAGCGAAATTCCCGAAGAAGAGGAGATTTATCGCACCATCAAACTGCCAGCAACAGTCAAGGAGGAAAATGCG
S E R I I Y S E I P E E E E I Y R T I K L P A T V K E E N A

TCTGCCAAATTCGAAAATGGCGTACTTTCGGTGATTCTGCCTAAAGCGGAAAGTTCCATCAAGAAAGGCATCAACATCGAA
S A K F E N G V L S V I L P K A E S S I K K G I N I E
