# Supplementary material for: A Study on the Temperature-Dependent Behavior of Small Heat Shock Proteins from Methanogens
Source: Int J Mol Sci. 2025 Jun 16;26(12):5748. doi: 10.3390/ijms26125748 (PMC12193508; doi:10.3390/ijms26125748)
Supplement: Supplementary file 1 [file ijms-26-05748-s001.zip › SupplementaryTable.pdf]

Supplementary Table

Primers used for making mutants

E43Q mutation in MMsHsp	
MM_E43Q_fw : GGGCTGCAGATTAGTGGCAAAGGCTTT	MM_E43Q_rv : ACTAATCTGCAGCCCCATGGAGTTCAT
Q36E mutation in MJsHsp	
MJ_Q36E_fw : GGTATTGAGATTTCGGGTAAAGGCTTT	MJ_Q36E_rv : CGAAATCTCAATACCCATGGAGGATTG
E59Q mutation in MMsHsp	
MJ_Q52E_fw : GGCGATGAACATATCAAAGTGATTGCG	MM_E59Q_rv : AATGGTCTGATCGCCTTCAATCAGGGT
Q52E mutation in MJsHsp	
MJ_Q52E_fw : GGCGATGAACATATCAAAGTGATTGCG	MJ_Q52E_rv : GATATGTTCATCGCCTTCAATGATGCT
D152N mutation in MMsHsp	
MM_D152N_fw : AGGCATTAACATTGAATAACTCGAGC	MM_D152N_rv : TCAATGTTAATGCCTGTGCGTTTGGC
N145D mutation in MJsHsp	
MJ_N145D_fw : AGGCATCGACATCGAATAACTCGAGC	MJ_N145D_rv : TCGATGTCGATGCCTTTCTTGATGGA
D152N mutation in MMsHsp	
MM_D152N_fw : AGGCATTAACATTGAATAACTCGAGC	MM_D152N_rv : TCAATGTTAATGCCTGTGCGTTTGGC
N145D mutation in MJsHsp	
MJ_N145D_fw : AGGCATCGACATCGAATAACTCGAGC	MJ_N145D_rv : TCGATGTCGATGCCTTTCTTGATGGA
E118G mutation in MJsHSp	
MJ_E118G_fw AAGGAGGGGAATGCGTCTGCCAAATTC	MJ_E118G_rv CGCATTCCCCTCCTTGACTGTTGCTG
A94M mutation in MMsHsp	
MM_3M_A94M_fw : CCTATGATGATCATGGAATCGGAGAA	MM_3M_A94M_rv : CATGATCATCATAGGTGCGCGTTTAGC
M96T mutation in MMsHsp	
MM_3M_M96T_fw : GCGATCACGGAATCGGAGAAAATCATC	MM_3M_M96T_rv : CGATTCCGTGATCGCCATAGGTGC
T89M mutation in MJsHsp	
MJ_3M_T89M_fw : ATGATTATGGAAAGTGAACGCATCA	MJ_3M_T89M_rv : ACTTTCCATAATCATGAGCGGAGAAC
MMsHsp-Chimera	
MM_3M_1_fw : ATCATCTATAGCGAAGTTCCGGAAGATGAAGAAGTCTACAAGACC<br>NMCJ_3M_1_rv : TTCGCTATAGATGATGCGTTCACTTTCCGTAATCATGAGCGGAGA<br>NMCJ_3M_2_fw : ATCATCTATAGCGAAATTCCCGAAGAAGAGGAGATTTATCGCACC<br>MM_3M_2_rv : TTCGCTATAGATGATTTCTCCGATTCCATGATCGCCATAGGTGC	
